# *Synurus deltoides* Alleviates Anti-Depressive Like Behavior Dysfunction Induced by Chronic Unpredictable Mild Stress *via* Stress-Related CRF/TLR Pathway

**DOI:** 10.4014/jmb.2501.01043

**Published:** 2025-05-15

**Authors:** Seung Gyum Joo, Jong Min Kim, Hyo Lim Lee, Min Ji Go, Tae Yoon Kim, Ju Hui Kim, Han Su Lee, Hyun Ji Eo, Hyun-Jin Kim, Ho Jin Heo

**Affiliations:** 1 Division of Applied Life Science (BK21), Institute of Agriculture and Life Science, Gyeonsang National University, Jinju 52828, Republic of Korea; 2 Korea Food Research Institute, Wanju-gun 55365, Republic of Korea; 3 Division of Special Forest Resources, Department of Forest Bio-resources, National Instiute of Forest Science, Seoul 0245, Republic of Korea

**Keywords:** *Synurus deltoides*, chronic unpredictable mild stress, depression, neuro-inflammation, hormonal pathway

## Abstract

This study was aimed at assessing the protective effect of the 80% ethanolic extract of *Synurus deltoides* (EESD) on chronic unpredictable mild stress (CUMS)-induced depressive-like behavior dysfunction. The bioactive compounds of *S. deltoides* were identified as quinic acid, chlorogenic acid, rutin, 1,3-dicaffeoylquinic acid, and dicaffeoylsuccinoylquinic acid. EESD and bioactive compounds in EESD significantly protected corticosterone-induced hippocampal cellular death and reactive oxygen species (ROS) contents compared to vitamin C in HT22 cells. By conducting the sucrose preference test, forced swimming test, open field test, and tail suspension test, EESD was found to significantly suppress depression-like behavior. EESD effectively reduced mitochondrial dysfunction by regulating cerebral ROS levels, mitochondrial membrane potential, and ATP contents. EESD showed a considerable regulatory effect by regulating serum stress hormones including corticosterone, norepinephrine, serotonin, 5-hydroxyindoleacetic acid, and melatonin. In addition, EESD significantly suppressed stress-related CRF pathway, inflammatory TLR pathway, and apoptotic signal in cerebral tissues. These results suggest that EESD might be a natural plant substance that improves CUMS-induced behavior abnormality by regulating inflammation and hormonal changes in brain tissue. In the future, additional clinical trials or efficacy evaluations of individual compounds of EESD will be needed to confirm the bioactivity ability and usability of EESD.

## Introduction

Stress is an inevitable aspect of human existence and has a negative impact on various areas of life [1]. The exposure of chronic stress extends beyond individual problems to encompass broader societal implications, manifesting in economic burdens and heightened disease prevalence [2]. Chronic exposure to stressors induces a significant challenge to human health and contributes to an increase in medical costs and diminishes overall body function [3]. At the physiological level, the complicated interaction between stress and the neuroendocrine system presents a complex pathway networks that modulate the body’s response to external stimuli [4]. The hypothalamic-pituitary-adrenal (HPA) axis serves as a pivotal conduit in orchestrating the body's reaction to stressors and regulating the secretion of cortisol as a key stress hormone [5]. Concurrently, stress has profound effects on neural structures, particularly brain regions associated with cognition and emotional regulation [6]. The complicated interaction between these systems is related to the profound influence of stress on neural function [7]. In particular, serotonin, a neurotransmitter vital for mood regulation, experiences dysregulation in response to stress, which predisposes individuals to mood disorders such as depression and anxiety [8]. The disruption of serotonin signaling cascades indicates the intricate interplay between stress and neuropsychiatric conditions and has wide consequences for mental health [9]. Moreover, stress-induced hormonal imbalances not only influence neurotransmitter activity but also precipitate inflammatory responses in the brain [10]. The dysregulation of stress hormones amplifies neuroinflammatory processes, culminating in heightened susceptibility to neurodegenerative disorders and aggravating existing psychiatric conditions [11]. In essence, chronic exposure to stressors stimulates a cascade of physiological and neurochemical alterations that predispose individuals to a spectrum of adverse health outcomes [12]. It is imperative to understand the intricate relationship between stress and health outcomes to develop targeted interventions aimed at mitigating the deleterious effects of stress on human health and well-being [13]. Therefore, it is important to prevent various symptoms that appear from chronic stress exposure.

*Synurus deltoides*, a species of *Asteraceae*, is a perennial plant found in many regions, including China, Japan, South Korea, and Russia [14]. The bioactive compounds found in various parts such as the roots and leaves of this plant mainly showed several physiological activities such as antioxidant, anti-inflammatory, antibacterial, and anticancer ones [15]. *S. deltoides* has strong antioxidant activity and anti-inflammatory effects and was found to be mainly mediated by phenolic compounds and other antioxidant substances in plants [16]. Its root extract has significantly high antioxidant capacity, which contributes to protecting cells from oxygen species by interacting with a variety of reaction mediators [14]. This mainly suppresses the expression of inflammatory mediators and inhibiting inflammation-related signaling pathways [16]. Additionally, the essential oil of *S. deltoides* also showed antibacterial activity [17]. Furthermore, some studies have shown that extracts of *S. deltoides* exhibit anticancer effects [18]. Therefore, additional study is necessary to confirm the physiological activity of this plant, especially to evaluate the exact mechanism and clinical applicability of its active compounds. On the other hand, there is a lack of studies that evaluate its protective effect on chronic stress exposure. Therefore, in this study, it was assessed to confirm the ameliorating effect of *S. deltoides*, which presented various physiological activities in chronic unpredictable mild stress (CUMS)-induced C57BL/6 mice.

## Materials and Methods

### Sample Preparation

*Synurus deltoides* leaves were purchased from Seosan-si (Republic of Korea) in May 2021 and verified by the National Institute of Forest Science (Republic of Korea). Dried sample extracted with 80% ethanol was filtered and concentrated. The extracted 80% ethanolic extract of *S. deltoides* (EESD) was dried and stored frozen at -20°C.

### Physiological Compound Analysis

To determine the bioactive compounds in EESD, the identification was analyzed using ultra performance liquid chromatography quadrupole-time of flight mass spectrometry (UPLC-Q-TOF/MS^E^, Xevo G2-S, Waters Corp., USA). The analysis conditions used for the electrospray ionization (ESI) source were performed according to previous study [19]. The chromatography was separated using C_18_ column (2.1 × 100 mm, 1.7 μm pore size). The mobile solvents were A (0.1% formic acid in distilled water) and B (0.1% formic acid in acetonitrile). The analysis conditions of the mobile gradient were as follows: 1% B at 0–1 min, 1–100% B at 1–7 min, 100% B at 7–8 min, 100–1% B at 8–8.2 min, and 1% B at 8.2–10 min. The separated compounds were analyzed using negative ESI, and details were as follows: ramp collision energy, 10–30 V; capillary voltage, 2.5 kV; cone voltage, 40 V; mass range, 50–1,500 *m/z*. UPLC system was analyzed by Waters Masslynx (Waters Corp.).

### Evaluation of Neuronal Protective Effect

**Cell culture and treatment.** HT22 cells obtained from the Department of Anatomy of the College of Veterinary Medicine, Gyeongsang National University (Republic of Korea), and incubated in DMEM medium with 10% FBS, 50 units/ml penicillin, and 100 μg/ml streptomycin in the conditions of 5% CO_2_ at 37°C.

To assess the cell viability, hippocampal HT22 cells (1 × 10^4^ cells/well) were treated with 10, 20, and 50 μg/ml of EESD, or 10, 50, and 100 μM quinic acid (30212, Cayman Chemical, USA), chlorogenic acid (70930, Cayman Chemical), rutin (19868, Cayman Chemical), or 1,3-dicaffeoylquinic acid (25022, Cayman Chemical). After 3 h, the corticosterone was treated and incubated for 24 h. Finally, each well was reacted with 3-(4,5-dimethylthiazol-2-yl)-2,5-diphenyltetrazolium bromide (MTT) solution (5 mg/ml) for 3 h. The MTT formazan were measured at a test wavelength of 570 nm and reference wavelength of 690 nm.

To assess the reactive oxygen species (ROS) content, hippocampal HT22 cells (1 × 10^4^ cells/well) were treated with 10, 20, and 50 μg/ml of EESD, or 10, 50, and 100 μM quinic acid, chlorogenic acid, rutin, or 1,3-dicaffeoylquinic acid. After 3 h, the corticosterone was treated and incubated for 24 h. Finally, each well was reacted with dichlorodihydrofluorescein diacetate (DCF-DA) solution (5 mg/ml) for 40 min. The fluorescence was measured at excitation wave 485 nm and emission wave 535 nm using a fluorometer (Infinite F200, Tecan, Switzerland).

### CUMS Procedures

The CUMS procedure was conducted according to a previous study [19]. The experimental animal was randomly exposed to 7 stresses, including cage swap, case tilting, empty case, food or water deprivation, mild restraint, overnight light exposure, and wet bedding, one per day for a week for 4 weeks. The cage swap was performed by exchanging their cage to another group for 24 h. The case tilting was performed by leaning at 45° for 24 h. The empty cage was performed by removing the bedding from the cage for 24 h. The food or water deprivation was performed by diverting the food or water for 24 h. The mild restraint was performed by placing mice into an acryl box (10.5 cm width × 10.5 cm length × 5.5 cm height) with breathing holes for 2 h. The overnight light exposure was performed by exposing mice to a light place. The wet bedding was performed by wetting the bedding with distilled water 24 h. The order of stresses was conducted using the Excel function (=randbetween) (Microsoft Office 2016, Microsoft Corp., USA).

### Animal Design

C57BL/6 mice (4-weeks, male) were purchased from animal supplier (Samtako, Republic of Korea). All animal experimental procedures followed the guidelines of the Animal Care and Use Committee of Gyeongsang National University (certificate: GNU-220314-M0030, approved on 14 March 2022) and performed by the Policy of the Ethical Committee of Ministry of Health and Welfare (Republic of Korea). Laboratory conditions were maintained as 22 ± 2°C, 55% humidity, and 12 h light/dark cycle. The experimental group were divided as follows. Normal control (NC) group (non-CUMS treatment with drinking water); CUMS (CUMS treatment with drinking water) group; EESD 20 group [CUMS treatment with EESD [20 mg/kg of body weight] administration] and EESD 50 group [CUMS treatment, EESD [50 mg/kg of body weight] administration]. EESD dissolved in drinking water was orally fed for each mice using a stomach tube once a day for 4 weeks. The doses (20 mg/kg and 50 mg/kg) were considered based on previous studies utilizing similar plant extracts, demonstrating efficacy within this range [20].

### Behavioral Tests

To measure the sucrose preference test (SPT), on the first day, each mouse was accommodated with two bottles containing 1% sucrose solution for 24 h. On the second day, one of sucrose bottles was changed to non-sucrose solution for 24 h. On the third day, to prevent their preference for sucrose location, the places of the two bottles (left/right) were changed and food and water were removed. Lastly, the consumption of each bottle was calculated.

Sucrose preference (%) = sucrose consumption/(sucrose consumption + water consumption) × 100

To assess the force swimming test (FST), the clear acrylic cylinder container was composed as 50 cm height × 20 cm diameter. The water depth was maintained at a level of 15 cm at 25 ± 1°C. Immobility and activity time of each mouse was evaluated by recording the movement for 6 min using a video tracking system (Smart 3.0, Panlab, Spain).

To assess the tail suspension test (TST), a white acrylic box (50 cm width × 50 cm length × 50 cm height) with a long iron rod placed on top of the acrylic box was used in the experiment. The mice's tail was placed in the center of the long iron rod, and their movement was recorded for 5 min using a video tracking system (Smart 3.0, Panlab).

To assess the open field test (OFT), open filed chamber was composed to a white acrylic box (50 cm width × 50 cm height × 50 cm length). Before the experiment, the mice were located the designated peripheral area. During test trial, the activity time of each mouse in the central (within 25 cm width × 25 cm length) area and peripheral areas was recorded for 5 min using a video tracking system (Smart 3.0, Panlab).

### Preparation of Brain Tissues

After behavior tests, brain and hypothalamus tissues were collected from the sacrificed mice. The brain tissue was used for mitochondrial activity and western blot analysis, and hypothalamus tissue was used for hormonal analysis.

### Mitochondrial Activity

Brain tissue was homogenized using cold mitochondria isolation (MI) buffer [215 mM mannitol, 75 mM sucrose, 0.1% bovine serum albumin [BSA], 20 mM HEPES sodium salt and 1 mM egtazic acid [EGTA], pH 7.2] to eliminate unbroken cells and nuclei. This homogenized tissue was centrifuged at 1,300 ×*g* for 5 min, and the obtained supernatant was centrifuged again at 13,000 ×*g* for 10 min to obtain pellets. An isolation buffer containing 1 mM EGTA and 0.1% digitonin was added to discard the synaptosomes and after 5 min, centrifuged at 13,000 ×*g* for 15 min at 4°C. The obtained pellets were mixed with isolation buffer and centrifuged at 10,000 ×*g* for 10 min. The final pellet was used for mitochondrial activity [21].

To assess the mitochondrial ROS, the isolated mitochondrial pellet was dissolved into KCl-based respiration buffer (125 mM potassium chloride, 2 mM potassium phosphate monobasic, 2.5 mM malate, 20 mM HEPES, 1 mM magnesium chloride, 5 mM pyruvate and 500 μM EGTA, pH 7.0) with 50 μM DCF-DA. After incubating for 2 min in the dark, the fluorescence was measured at excitation wave 485 nm and emission wave 535 nm using a fluorometer (Infinite F200, Tecan, Switzerland).

To investigate the mitochondrial membrane potential (MMP), the isolated mitochondrial pellet with MI buffer, containing 5 mM pyruvate and 5 mM malate, was reacted with 1 μM 5,5,6,6-tetrachloro-1,1,3,3-tetraethyl-benzimidazolylcarbocyanine iodide. After incubating for 2 min in the dark, the fluorescence was measured at excitation wave 530 nm and emission wave 590 nm using a fluorometer (Infinite F200, Tecan).

To measure the mitochondrial ATP content, the isolated mitochondrial pellet centrifuged at 13,000 ×*g* for 10 min was mixed with 1% trichloroacetic acid for 10 min. After mixing with 25 mM tris-acetate buffer (pH 7.7) at 10,000 ×*g* for 15 min, the supernatant was used for the ATP content using an ATP commercial kit (Promega Corp., USA) using a luminometer (GloMax-Multi Detection System, Promega Corp.). The mitochondrial ATP contents were calculated according to standard curve.

### Hormonal Analysis

Hypothalamic hormonal metabolites were analyzed using an ultraperformance liquid chromatography ion mobility separation-quadrupole time of flight/tandem mass spectrometry (UPLC-Q-TOF/MS^E^, Waters Corp.) with positive ESI mode. MRM conditions were presented in Table 1.

### Western Blot Analysis

Proteins were extracted from the brain tissue using a lysis buffer (ProtinEx Animal cell/tissue, Gene All Biotechnology Co. Ltd., Republic of Korea) with 1% protease inhibitor. The extracted protein was centrifuged at 13,000 ×*g* for 10 min. Each sample was separated by sodium dodecyl sulfate polyacrylamide gel electrophoresis (SDS-PAGE) and transferred to a poly-vinylidene difluoride membrane. The membrane with transferred proteins was blocked using 5% skim milk for 1 h. The primary antibody was reacted, and a secondary antibody was reacted. The membrane was reacted with ECL (ProNA ECL Ottimo, TransLab., Republic of Korea), and the expression density was detected using iBrightTM CL1000 instrument (Invitrogen, USA). The obtained density was calculated using ImageJ software (National Institutes of Health, USA). The antibody information is presented in Table 2.

### Statistical Analysis

All results were repeated (*n* = 3) and expressed as mean ± standard deviation. Each mean value was analyzed for variance followed by Tukey’s post-hoc analysis using SAS version 9.4 (SAS institute, USA). Additionally, a univariate analysis of covariance (ANCOVA), with the baseline as a covariate, was used to compare the post-treatment values.

## Results

### Physiological Compound Using UPLC Q-TOF/MS^E^

**Fig. 1:**
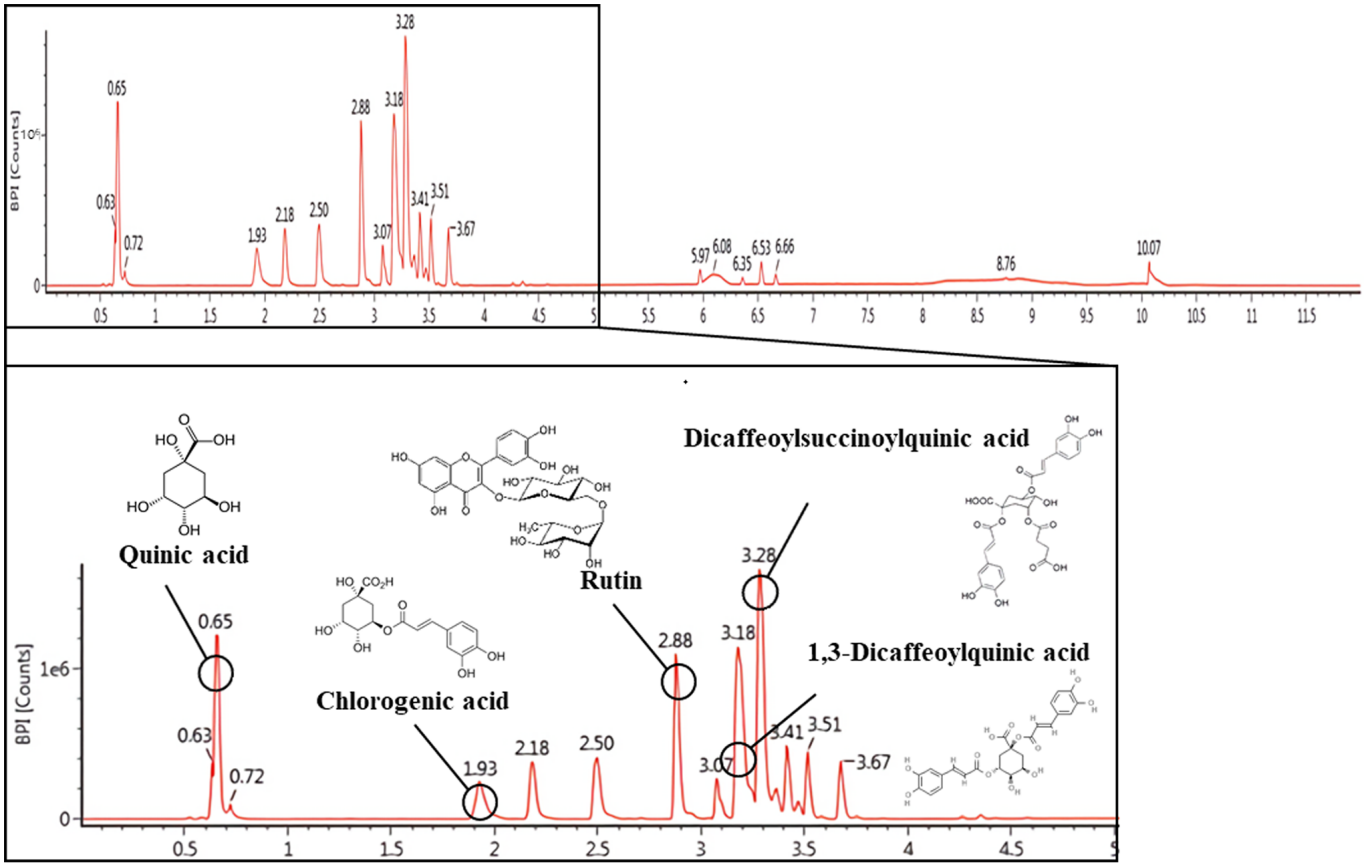
UPLC Q–TOF/MS chromatography of ethanolic extract of *Synurus deltoides*.

The bioactive compounds of EESD were identified using UPLC-Q-TOF/MS^E^ analysis (Fig. 1 and Table 3). The spectra of each compound obtained in negative ion mode was determined as compound 1, 191 *m/z* (retention time (RT): 0.65 min); compound 2, 353 *m/z* (RT: 1.93 min); compound 3, 609 *m/z* (RT: 2.88 min); compound 4, 515 *m/z* (RT: 3.18 min); compound 5, 615 *m/z* (RT: 3.28 min). These compounds were tentatively identified as quinic acid (compound 1), chlorogenic acid (compound 2), rutin (compound 3), 1,3-dicaffeoylquinic acid (compound 4) and dicaffeoylsuccinoylquinic acid (compound 5) according to a library software program (Waters Masslynx, Waters Corp.) and previous studies [22-25].

### Neuroprotective Effect

**Fig. 2:**
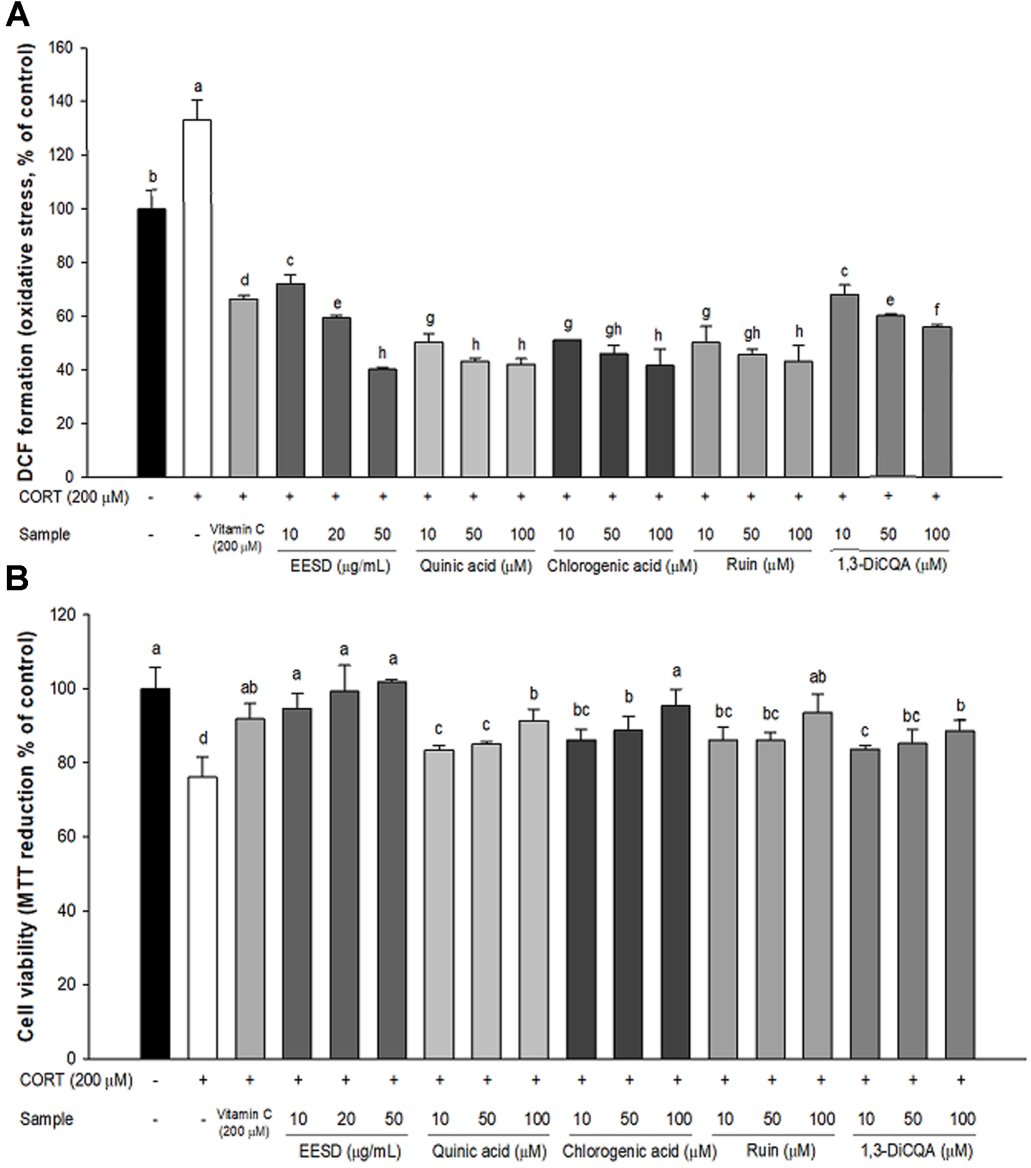
Neuroprotective effects of ethanolic extract of *Synurus deltoides* (EESD) and bioactive compounds, including quinic acid, chlorogenic acid, rutin, and 1,3-dicaffeoylquinic acid (1,3-DiCQA) in EESD in HT22 cells. corticosterone−induced ROS content (**A**) and cell viability (**B**). Results shown are mean ± SD (*n* = 5). Data are statistically represented at *p* < 0.05, and different lowercase letters indicate statistical significance. Means with the same letter are not significantly different.

The cell viability of the corticosterone-induced group (76.04%) was reduced compared to the normal control group (100%) (Fig. 2A). However, that of the vitamin C and EESD-treated groups was increased (vitamin C, 91.79%; 10 μg/ml, 94.68%; 20 μg/ml, 99.25%; 50 μg/ml, 101.92, respectively) compared to the corticosterone-induced group. In addition, to evaluate the protective effect of each compound in EESD, the cell viabilities of quinic acid, chlorogenic acid, rutin, and 1,3-dicaffeoylquinic acid were analyzed. Dicaffeoylsuccinoylquinic acid, which was identified in isomer form, was excluded from the experiment. Cell viabilities of the 50 μM each compound (quinic acid, 91.28%; chlorogenic acid, 35.32%; rutin, 93.52%; 1,3-dicaffeoylquinic acid, 88.51%)-treated group were increased compared to the corticosterone-induced group. The reactive oxygen species (ROS) contents of the corticosterone-induced group (132.84%) were increased compared to the normal control group (100%) (Fig. 2B). However, that of the vitamin C and EESD-treated groups decreased (vitamin C, 66.40%; 10 μg/ml, 72.13%; 20 μg/ml, 59.39%; 50 μg/ml, 40.12%, respectively) compared to the corticosterone-induced group. In addition, the ROS contents of the 50 μM each compound (quinic acid, 42.09%; chlorogenic acid, 42.62%; rutin, 43.06%; 1,3-dicaffeoylquinic acid, 55.82%)-treated group were decreased compared to the corticosterone-induced group.

### Behavioral Test

**Fig. 3:**
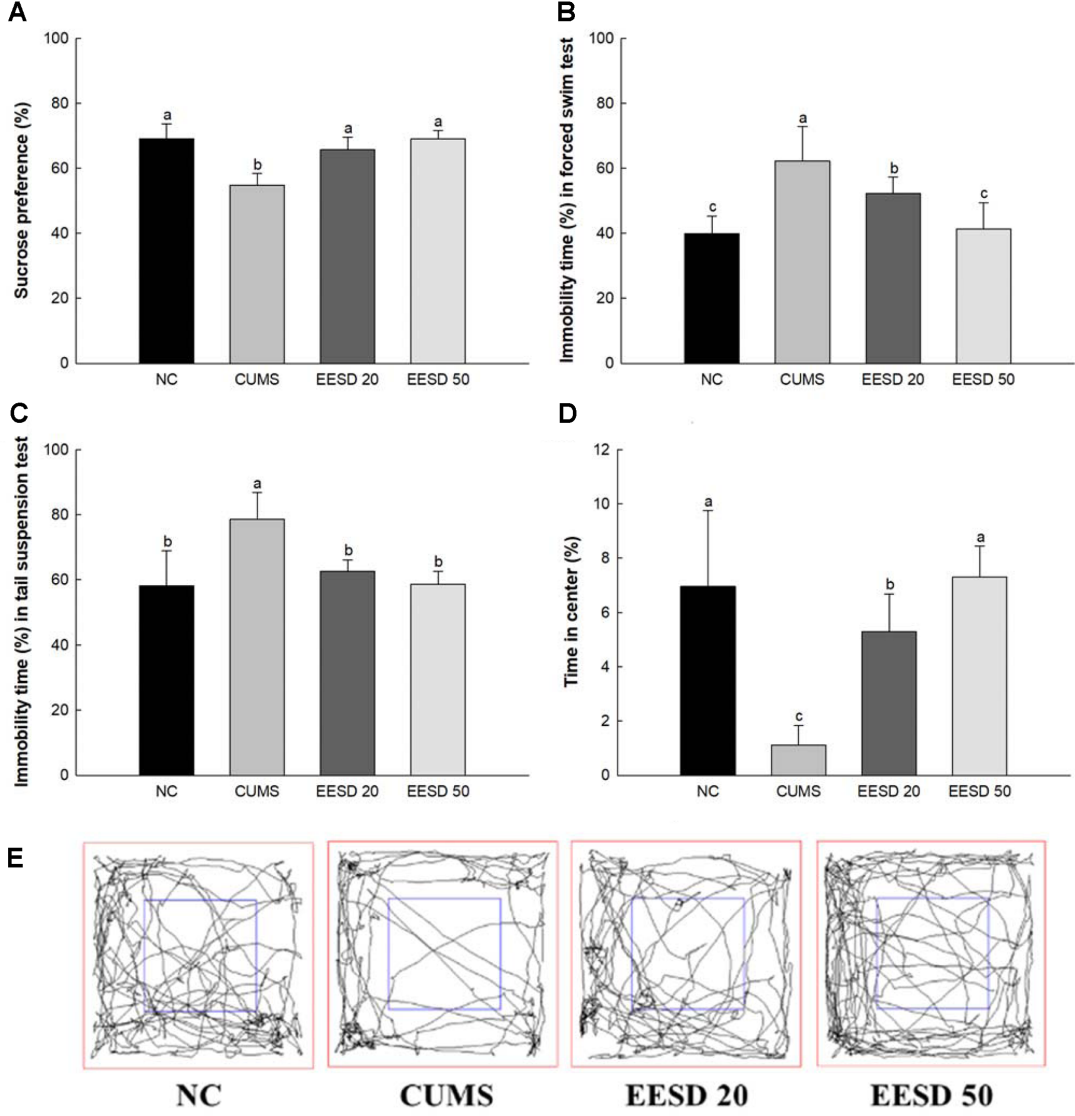
Effects of ethanolic extract of *Synurus deltoides* (EESD) on depression-like behaviors in CUMSinduced mice. Sucrose preference in sucrose preference test (SPT) (**A**) immobility time in forced swimming test (FST) (**B**) immobility time in tail suspension test (TST) (**C**) probe trial (**D**) in open field test (OFT), and ratio of center zone in OFT (**E**). Results shown are mean ± SD (*n* = 5). Data are statistically represented at *p* < 0.05, and different lowercase letters indicate statistical significance. Means with the same letter are not significantly different.

In the results of the SPT of the CUMS group (54.76%), the sucrose preference ratio was reduced compared with the NC group (69.06%) (Fig. 3A). However, that of the EESD groups (EESD 20, 65.65%; EESD 50, 69.05%, respectively) was increased compared to the CUMS group (Fig. 3A). In the results of the FST of the CUMS group (62.32%), the immobility time was significantly increased compared to the NC group (39.90%) (Fig. 3B). On the other hand, for the EESD groups (EESD 20, 52.25%; EESD 50, 41.39%, respectively), it was reduced compared to the CUMS group (Fig. 3B). In the results of the TST of the CUMS group, the immobility time (78.51%) was significantly increased compared to the NC group (58.19%) (Fig. 3C). On the other hand, for the EESD group (EESD 20, 62.52%; EESD 50, 58.62%, respectively), it was decreased compared to the CUMS group. In the results of the OFT of the CUMS group the time spent in the center zone (1.11%) was decreased compared to the NC group (6.95%) (Fig. 3D and 3E). However, for the EESD groups (EESD 20, 5.30%; EESD 50, 7.29%, respectively) it was increased compared to the CUMS group.

### Mitochondrial Activity

**Fig. 4:**
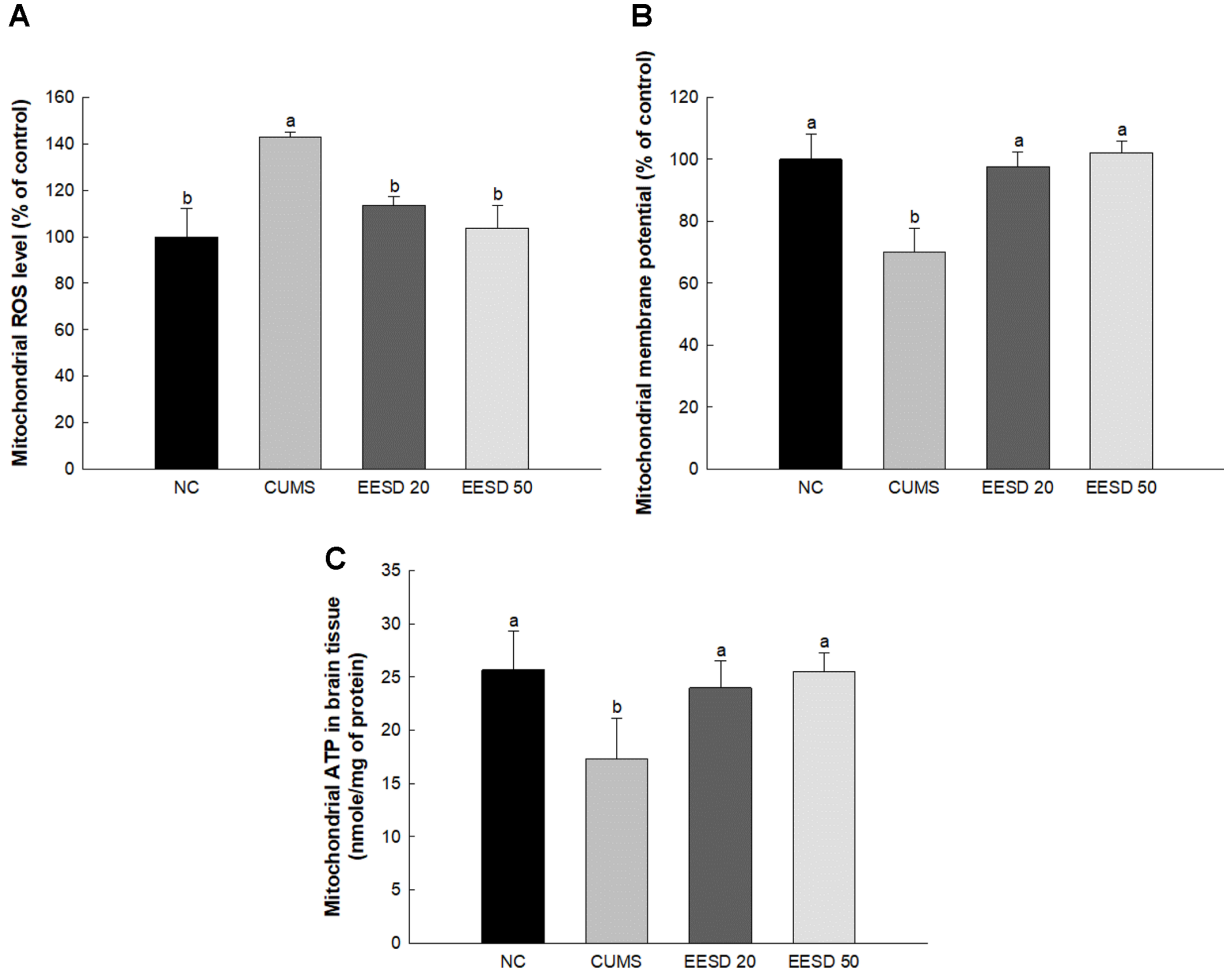
Effect of ethanolic extract of *Synurus deltoides* (EESD) on CUMS-induced mitochondrial dysfunction. Mitochondrial ROS contents (**A**) mitochondrial membrane potential (MMP) contents (**B**) and ATP contents (**C**). Results shown are mean ± SD (*n* = 5). Data are statistically represented at *p* < 0.05, and different lowercase letters indicate statistical significance. Means with the same letter are not significantly different.

The ROS content in the CUMS group (142.85%) was increased compared to the NC group (100%) (Fig. 4). However, that of the EESD groups (EESD 20, 113.47%; EESD 50, 103.64%, respectively) was decreased compared to the CUMS group. The MMP of the CUMS group (70.01%) was decreased compared with the NC group (100%). However, that of the EESD groups (EESD 20, 97.71%; EESD 50, 102.09%, respectively) were increased compared to the CUMS group. The ATP levels of the CUMS group (17.29 nmole/mg of protein) increased compared to the NC group (25.66 nmole/mg of protein). However, those of the EESD groups (EESD 20, 23.98 nmole/mg of protein; EESD 50, 25.51 nmole/mg of protein, respectively) were increased compared to the CUMS group.

### Hypothalamic Hormone Change

**Fig. 5:**
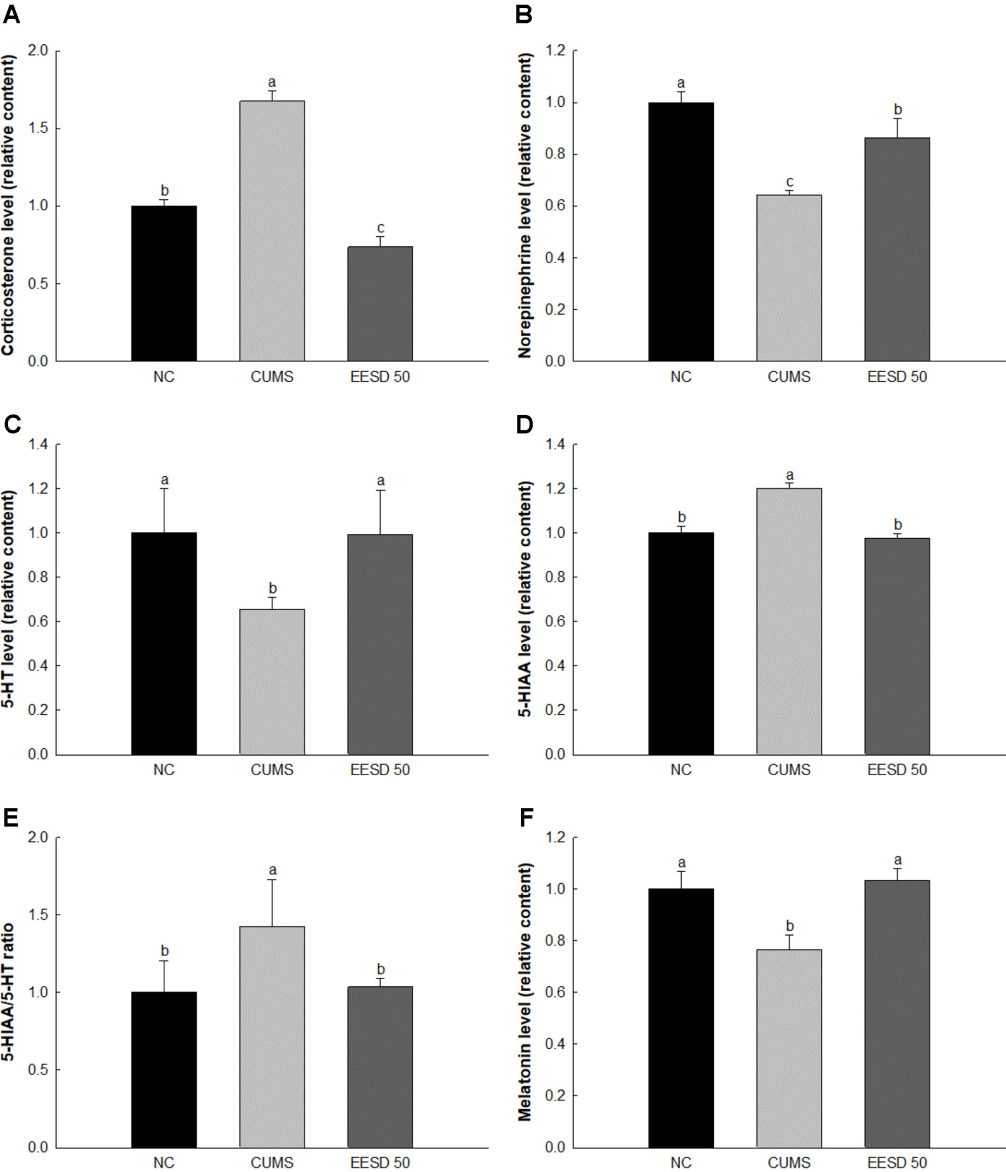
Effect of ethanolic extract of *Synurus deltoides* (EESD) on corticosterone (A) norepinephrine (B) 5- hydroxytryptamine (5-HT; serotonin) (C) 5-hydroxyindoleacetic acid (5-HIAA) (D) 5-HIAA/5-HT ratio (E) and melatonin (F) contents in hypothalamus. Results shown are mean ± SD (*n* = 3). Data are statistically represented at *p* < 0.05, and different lowercase letters indicate statistical significance. Means with the same letter are not significantly different.

Subsequent experiments focused on the EESD 50 group, where the most pronounced neuroprotective effects were observed. Hypothalamic corticosterone and 5-hydroxyindoleacetic acid (5-HIAA) levels of the CUMS group (167.36%, and 120.10%, respectively) were increased compared to the NC group (100%) (Fig. 5). However, those of the EESD 50 group (73.59% and 97.60%, respectively) were decreased compared to the CUMS group. Hypothalamic norepinephrine, 5-hydroxytryptamine (serotonin, 5-HT), and melatonin levels of the CUMS group (64.16%, 65.64%, and 76.65%, respectively) were reduced compared to the NC group (100%). However, those of the EESD 50 group (86.49%, 99.38%, and 103.20%, respectively) were increased compared to the CUMS group.

### Stress-Related Pathway

**Fig. 6:**
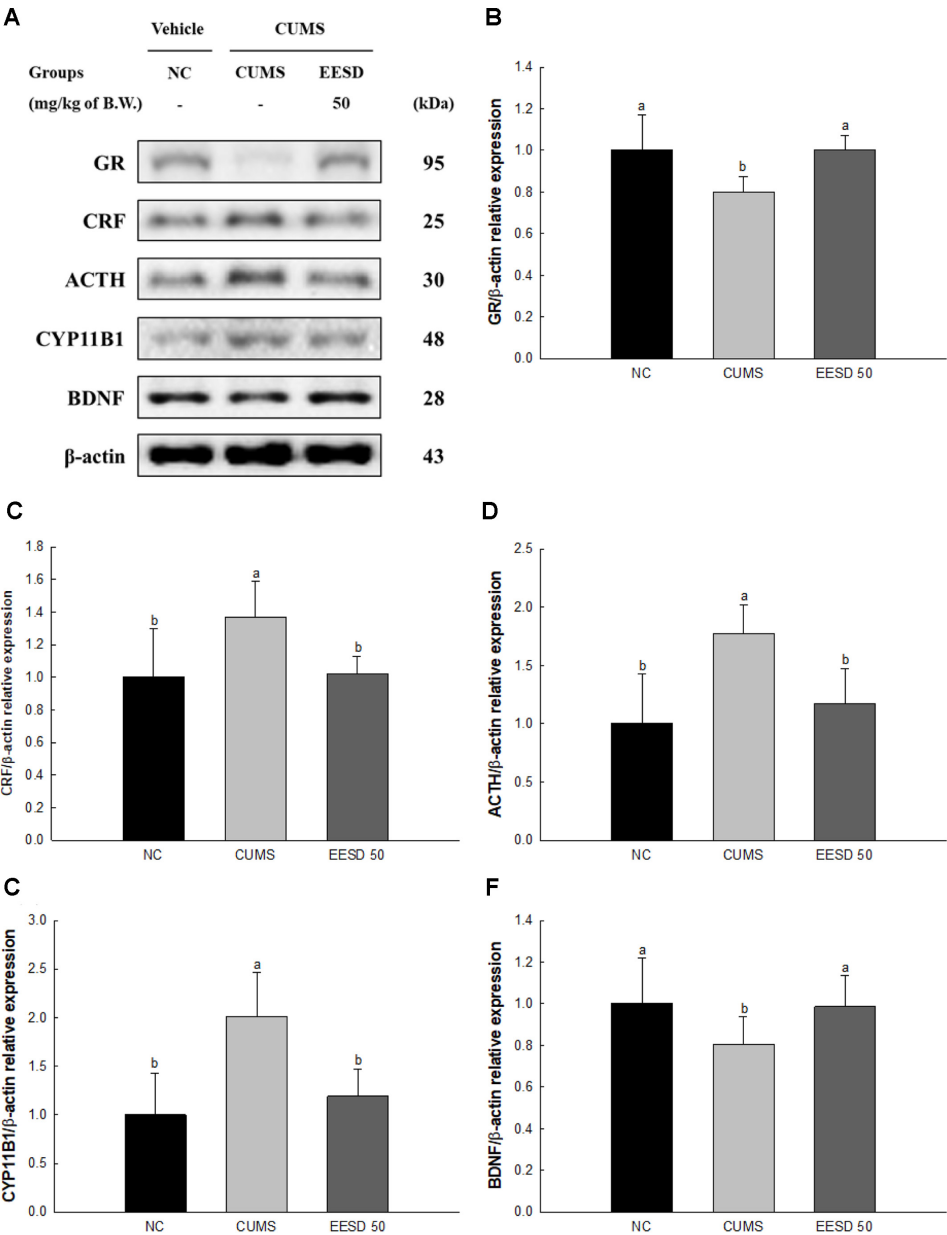
Effect of ethanolic extract of *Synurus deltoides* (EESD) on CUMS-induced stress pathway in brain tissue. Western blot images (**A**) protein expression levels of GR (**B**) CRF (**C**) ACTH (**D**) CYP11B1 (**E**) and BDNF (**F**) in brain tissues. Results shown are mean ± SD (*n* = 3). Data are statistically considered at *p* < 0.05, and different lowercase letters indicate statistical significance. Means with the same letter are not significantly different.

The protein expression of the glucocorticoid receptor (GR) (79.87%) and brain-derived neurotrophic factor (BDNF) (80.68%) in the CUMS group were downregulated compared to those in the NC group (100%) (Fig. 6). However, the EESD 50 group statistically upregulated GR (100.27%) and BDNF (98.49%) compared to the CUMS group. The protein expression of corticotropin-releasing hormone (CRF) (136.99%), adrenocorticotropic hormone (ACTH) (177.26%) and cytochrome P450 Family 11 Subfamily B Member 1 (CYP11B1) (201.06%) in the CUMS group were upregulated compared to those in the NC group (100%). However, the EESD 50 group downregulated CRF (102.11%), ACTH (117.24%) and CYP11B1 (119.07%) compared to the CUMS group.

### Protein Expression of Neuro-Inflammation

**Fig. 7:**
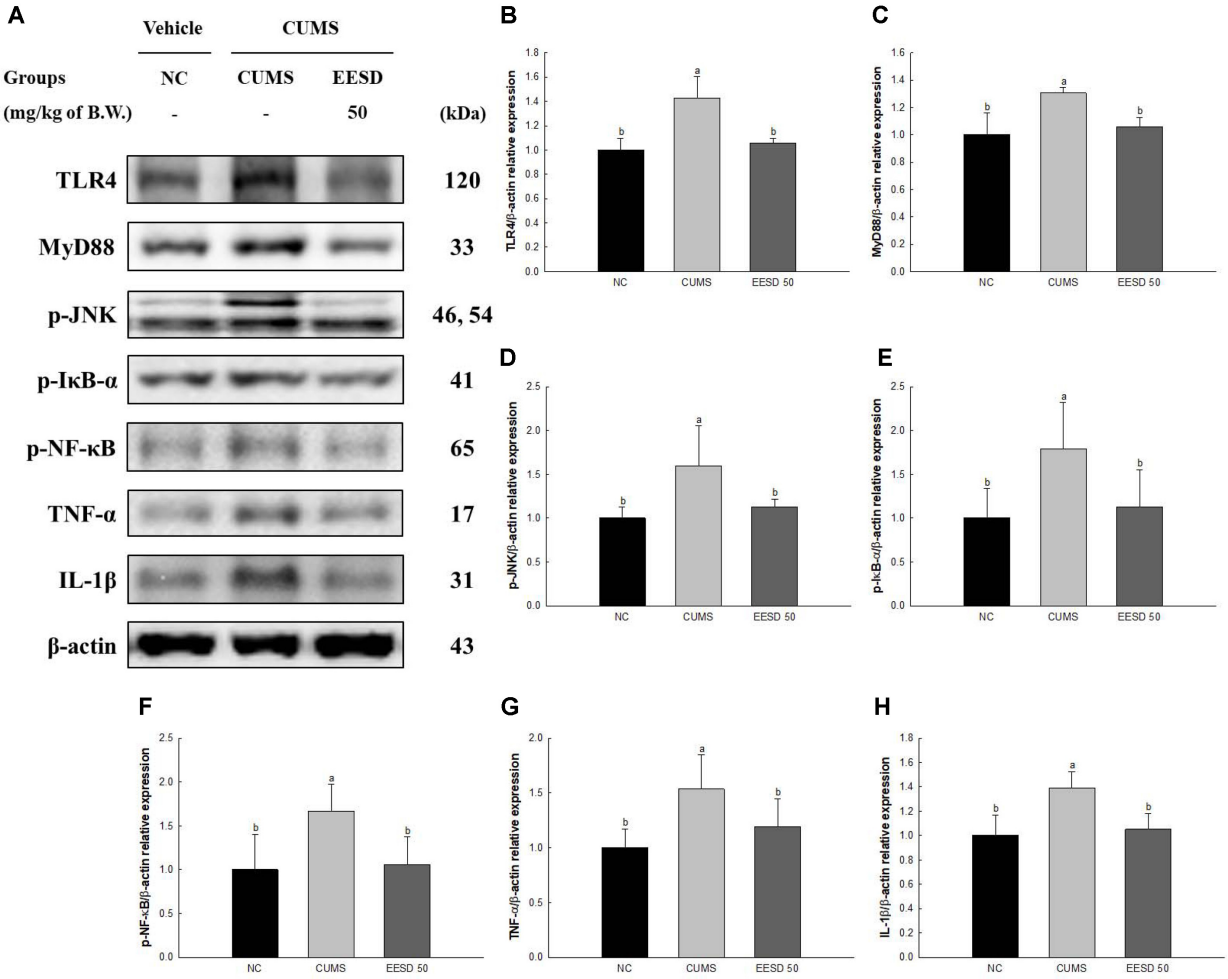
Effect of ethanolic extract of *Synurus deltoides* (EESD) on CUMS-induced stress pathway in brain tissue. Western blot images (**A**) protein expression levels of TLR4 (**B**) MyD88 (**C**) p-JNK (**D**) p-IκB-α (**E**) p-NF-κB (**F**) TNF-α (**G**) and IL-1β (**H**) in brain tissues. Results shown are mean ± SD (*n* = 3). Data are statistically considered at *p* < 0.05, and different lowercase letters indicate statistical significance. Means with the same letter are not significantly different.

The protein expression of Toll-like receptor 4 (TLR4, 142.90%), myeloid differentiation primary response 88 (MyD88, 130.54%), phosphorylated c-Jun *N*-terminal kinases (p-JNK, 159.79%), phosphorylated nuclear factor of kappa light polypeptide gene enhancer in B-cells inhibitor alpha (p-IκB-α, 178.92%), phosphorylated nuclear factor kappa-light-chain-enhancer of activated B cells (p-NF-κB, 166.59%), tumor necrosis factor-α (TNF-α, 153.55%) and interleukin-1 beta (IL-1β, 139.10%) of the CUMS group were upregulated compared to the NC group (100%) (Fig. 7). However, the EESD groups decreased the protein expression of TLR4 (105.84%), MyD88 (105.97%), p-JNK (112.82%), p-IκB-α (113.14%), p-NF-κB (106.11%), TNF-α (119.30%) and IL-1β (105.10%) compared to the CUMS group.

### Protein Expression of Apoptosis

**Fig. 8:**
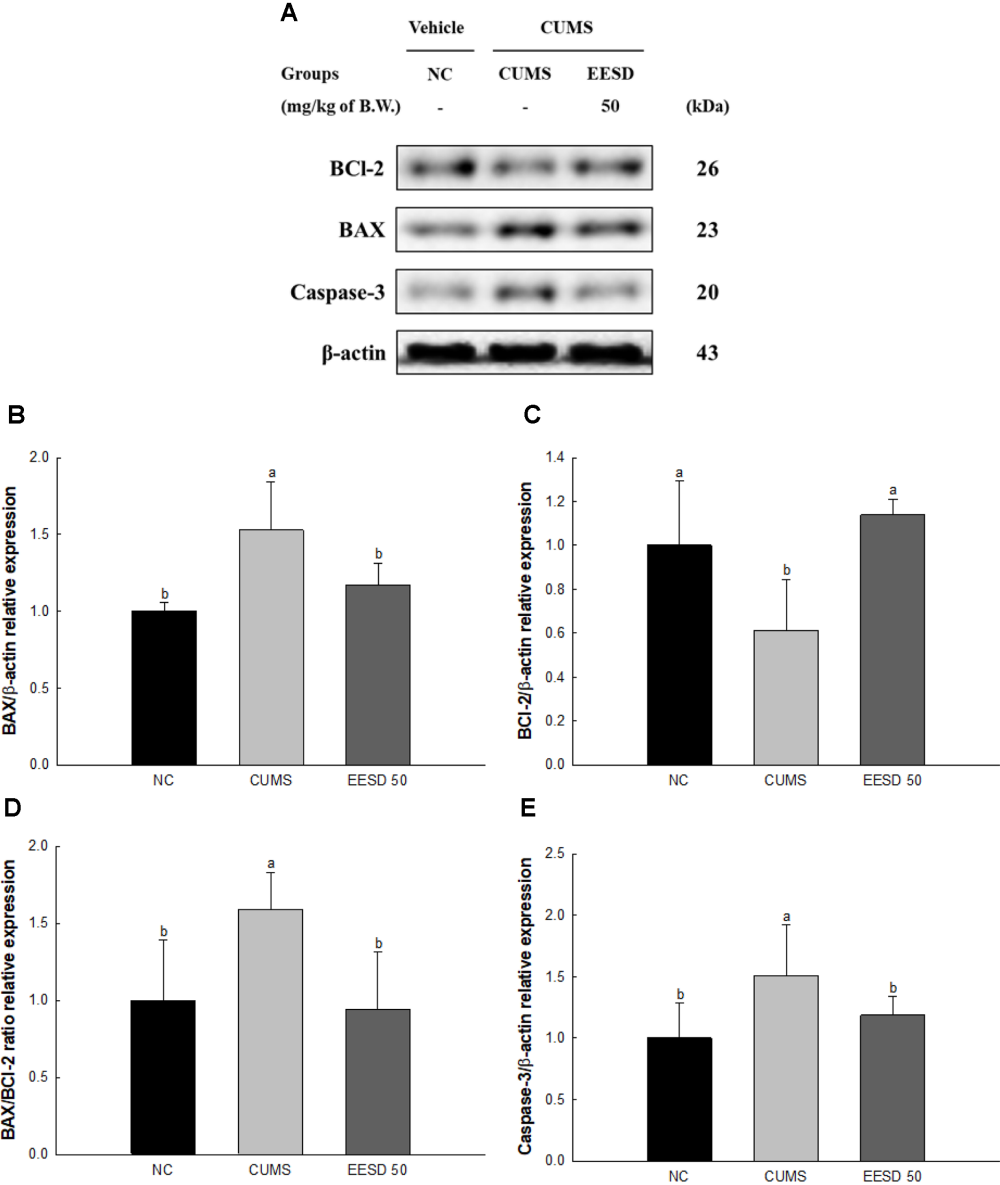
Effect of ethanolic extract of *Synurus deltoides* (EESD) on CUMS-induced stress pathway in brain tissue. Western blot images (**A**) protein expression levels of BCl-2 (**B**) BAX (**C**) BAX/BCl-2 ratio (**D**) and caspase-3 (**E**) in brain tissues. Results shown are mean ± SD (*n* = 3). Data are statistically considered at *p* < 0.05, and different lowercase letters indicate statistical significance. Means with the same letter are not significantly different.

The protein expression of B-cell lymphoma 2 (BCl-2) (61.14) of the CUMS group was downregulated compared to the NC group (100%) (Fig. 8). However, the protein expression of BCl-2 (113.99%) of the EESD groups was upregulated compared to the CUMS group. The protein expression of BCl-2-associated X protein (BAX)(152.87%) and caspase-3 (150.85%) of the CUMS group was upregulated compared to the NC group (100%). However, the protein expression of BAX (117.20%) and caspase-3 (118.71%) of the EESD groups was upregulated compared to the CUMS group.

## Discussion

Chronic exposure to stress is reported to cause a variety of damage in the body and especially affects brain tissue [3]. Excessive stimulation that is difficult for the body to tolerate promotes the secretion of various stress hormones and damages homeostasis in the body [9]. These reactions damage the immune system, stimulate inflammatory responses, and lead to psychological problems such as depression [5]. Therefore, a study was conducted to confirm the improvement effect of *S. deltoides* extract on stress stimulation and to identify the improvement mechanism.

Chronic stress disrupts serotonin neurotransmission and increases cortisol levels, contributing to anxiety and cognitive impairments [26, 27]. In this study, EESD administration significantly ameliorated depression-like behaviors, as evidenced by improvements in the SPT, FST, and TST (Fig. 3). These behavioral changes have a negative impact on an individual's daily functioning and quality of life due to the ongoing effects of stress [10]. This study evaluated whether EESD consumption has protective effects against depression-like behavior function by SPT, FST, and TST, and it significantly reduced behavioral abnormalities (Fig. 3). Similar to previous study, rutin, a major physiological compound in EESD, significantly ameliorated behavioral dysfunction with the regulation of the HPA axis [28]. Chlorogenic acid also suppressed depressant activity by regulating the BDNF/tropomyosin receptor kinase B (TrkB) pathway [29]. Dicaffeoylquinic acid showed an anti-depressive effect with the regulation of 5-HT and CORT levels [30, 31]. This suggests that the various phenolic compounds contained in EESD might help improve depression by promoting hormonal changes through HPA axis regulation. Although EESD exhibited more substantial neuroprotective effects than its individual active compounds, this study did not directly assess potential synergistic interactions between these components or quantify their exact concentrations. Future investigations should evaluate whether the observed efficacy is due to true synergy or an additive effect by comparing individual compounds' neuroprotective effects and combinations.

Hormonal imbalances affect the energy metabolism of neurons, where mitochondria produce energy [32]. Cortisol and other stress hormones, which increase during stressful situations, regulate the activity of mitochondria in neuronal cells, thereby affecting energy production processes [33]. Excessive secretion of these hormones causes neuronal energy metabolism to become irregular and reduces mitochondrial function [34]. Additionally, hormonal imbalances caused by chronic stress induce oxidative stress [35]. This dysfunction reduces cell survival and function in brain tissue and leads to more serious neurological abnormalities [36]. Therefore, chronic stress exposure reduces mitochondrial function, as hormonal imbalances affect neuronal energy metabolism, generate oxidative stress, and cause mitochondrial dysfunction [37]. *S. deltoides* contains many flavonoid compounds such as apigenin, myricetin, cyanidin, epicatechin, and catechin [15]. These flavonoids have strong antioxidant activity and a scavenging effect on oxidative stresses that generate ROS in neuronal mitochondria [38]. In particular, ROS scavenging activity in brain tissue protects the function of neuronal cells and improves energy metabolism abnormality, which might help improve mitochondrial disorders induced by chronic stress [39]. EESD exhibited mitochondrial protective effects against CUMS by regulating mitochondrial ROS contents, MMP levels and mitochondrial ATP contents (Fig. 4). Mitochondrial impairment induced by CUMS initially arises due to the increased generation of ROS, which interact with cellular molecules, compromising mitochondrial function [40]. However, a variety of compounds in EESD such as quinic acid and chlorogenic acid have antioxidant and anti-inflammatory properties [41, 42]. Dicaffeoylquinic acid and chlorogenic acid, in particular, protected mitochondria by scavenging free radicals and ROS generation [43]. Moreover, rutin inhibits inflammatory responses and enhances cellular oxygen supply, thereby improving mitochondrial function [43, 44]. These compounds maintain mitochondrial function and protect defense mechanisms against ROS, as a result alleviating CUMS-induced mitochondrial damage. Consequently, the protective effects of *S. deltoides* against CUMS might be mediated by the actions of compounds that reduce mitochondrial damage.

Chronic stress can negatively alter hormone metabolism processes in the body [9]. These changes occur through various pathways. Firstly, stress affects the HPA axis, regulating hormone secretion and leading to fluctuations in blood hormone levels [4]. In stress situations, cortisol and other glucocorticoid hormones are secreted from the adrenal glands and influence neurotransmitter regulation and modulate stress responses and coping mechanisms [26]. The serotonin metabolic pathway is also influenced by chronic stress [8]. Chronic stress reduces serotonin levels and disrupts signaling associated with mood regulation [45]. Additionally, glucocorticoids affect important cell signaling molecules such as glycerol, which is linked to serotonin level reduction [46]. Hormonal changes have direct effects on brain tissue [45]. Persistent elevation of glucocorticoids by chronic stress causes neuro-inflammation and hormonal imbalance in the hippocampus, leading to structural changes and impairments in its function [47]. The hippocampus, crucial for memory and learning, may suffer from reduced functionality, resulting in memory deficits and decreased learning abilities [48]. Moreover, increased glucocorticoid levels due to chronic stress promote brain inflammation, which may cause damage to brain tissue and even neuronal injury and cell death [49]. Chronic stress stimulates abnormal metabolic pathways in the brain and influences factors such as CRF, ACTH, CYP11B1, and BDNF [50]. CRF, a neuropeptide produced in the hypothalamus, plays a crucial role in initiating the stress response by stimulating the secretion of ACTH from the pituitary gland [51]. Continuous secretion of glucocorticosterone disrupts the normal function of CRF, leading to excessive stress responses such as ACTH and cytokines secretion [50]. ACTH continuously stimulates the adrenal glands to produce glucocorticoids [52]. Chronic stress dysregulates the production and release of ACTH, resulting in altered glucocorticoid levels [53]. Additionally, this abnormal mechanism influences the expression of CYP11B1, an enzyme involved in glucocorticoid synthesis, further exacerbating the dysregulation of glucocorticoid metabolism [50]. In addition, chronic exposure to elevated corticosterone levels can lead to negative feedback downregulation of GR expression [54]. Prolonged GR activation may result in receptor desensitization and reduced receptor availability, which has been observed in various chronic stress models, and contribute to impaired HPA axis regulation and stress-related neuronal dysfunction [55, 56]. Through this pathway, chronic stress continuously stimulates the production of glucocorticosterone, resulting in a decline in the function of neuro-transmitters and BDNF in brain tissue [57]. Furthermore, chronic stress suppresses the expression of BDNF, a protein crucial for neuronal survival, growth, and synaptic plasticity, and causes mood disorders such as depression [58]. These alterations cause structural and functional changes in the brain, including reduced neurogenesis, synaptic remodeling, and altered neurotransmitter systems, which are hallmark features of depression [59]. Overall, chronic stress disrupts cerebral metabolic pathways and affects factors such as glucocorticosterone, CRF, ACTH, CYP11B1, and BDNF [50]. These disruptions induce the pathophysiology of depression by causing structural and functional changes in the brain, between stress, brain metabolism, and mood disorders [60]. *S. deltoides* contains a variety of compounds that might contribute to protective effects against CUMS by regulating HPA axis hormones and neurotransmitters. Quinic acid, rutin, and other bioactive compounds act synergistically to counteract the effects of chronic stress on the HPA axis and neurotransmitter systems [61]. Quinic acid and chlorogenic acid reduce oxidative stress and inflammation, thereby protecting neuronal cells from damage induced by stress hormones such as cortisol [62, 63]. Rutin, in addition, has been found to modulate serotonin levels and enhance serotonin receptor activity, which contributes to its antidepressant and anxiolytic effects [64]. Furthermore, *S. deltoides* extract has been shown to regulate the expression of proteins involved in stress signaling pathways, including GR, BDNF, and CRF (Fig. 6). These mechanisms show the ability of *S. deltoides* to attenuate physiological and behavioral responses to chronic stress. Therefore, *S. deltoides* exerts its protective effects against CUMS through its modulation of HPA axis hormones and neuro-transmitters, including serotonin and cortisol. The diverse bioactive compounds in *S. deltoides* might synergistically regulate stress-related pathways and mitigate the depressive effects of chronic stress on physiological and neurochemical systems.

Chronic stress stimuli activate inflammatory responses in brain tissue through various pathways [65]. Chronic stress-induced activation of the CRF pathway stimulates the TLR4 pathway, leading to inflammatory responses in brain tissue [66]. This pathway involves several key subfactors including MyD88, TIR-domain-containing adapter-inducing interferon-β (TRIF), NF-κB, and mitogen-activated protein kinases (MAPKs) [67]. MyD88 serves as a major protein mediating signal transduction from TLR4, and interacting with TLR4 to regulate inflammatory responses through NF-κB and MAPKs [68]. NF-κB regulates the expression of inflammatory genes, activated through the CUMS-induced TLR4 pathway to promote the expression of inflammatory cytokines [69,70]. Inflammatory responses stimulated by these negative pathways lead to changes in various inflammatory cytokines and apoptosis cascades [34]. Inflammatory cytokines, such as IL-1β, TNF-α, IL-6, IL-8, and IL-12, promote inflammation in brain tissue and induce a variety of behavioral changes [71]. Additionally, inflammatory cytokines disrupt the balance of neurotransmitter systems in the brain, leading to emotional disturbances, and damage interactions with neurotransmitter systems. They also alter stress response mechanisms and promote the onset of stress-related disorders [66]. Therefore, chronic stress triggers inflammatory responses in brain tissue, leading to a variety of behavioral changes [72]. The protective effect of EESD against CUMS is closely associated with the expression of inflammation and cytokines in neuronal cells. However, compounds in *S. deltoides*, such as apigenin, myricetin, cyanidin, epicatechin, and catechin, contribute to the protection of neuronal cells by suppressing these inflammatory responses [15]. These compounds inhibit the activation of NF-κB and MAPK pathways via TLR4 and MyD88, thereby suppressing inflammatory responses [73, 74]. Additionally, they inhibit the expression of pro-inflammatory cytokines such as TNF-α and IL-1β, thus protecting neuronal cells [74, 75]. Furthermore, these compounds might protect against neuronal cell damage and regulate inflammatory responses by enhancing the neuropro-tective effects of EESD [76]. In addition, *S. deltoides* (Aiton) Nakai considerably inhibited the production of nitric oxide (NO), prostaglandin E2 (PGE2), and expression levels of COX-2 and TNF-α via activation protein (AP)-1 and the NF-κB pathway [16]. In summary, the bioactive compounds in *S. deltoides* regulate neuronal cells by inhibiting inflammation and cytokine expression induced by CUMS.

Mitochondrial dysfunction and death are highly associated with chronic stress [31]. In stressful situations, the secretion of stress hormones such as cortisol increases, which can negatively affect mitochondrial function [33]. Mitochondria receive and regulate hormone signals through various receptors, including hormone receptors, and changes in hormone receptors during stress situations lead to mitochondrial dysfunction [35]. In addition, changes in various mitochondrial hormone receptors are induced by stress [77]. Mitochondria contain a variety of hormone receptors, including estrogen receptors, progesterone receptors, and testosterone receptors [78]. The levels of these receptors change, which can affect mitochondrial function, induce mitochondrial dysfunction and apoptosis by inducing oxidative stress, changes in mitochondrial hormone receptors, and apoptosis [79]. One of the key pathways involved in apoptosis is the BCl-2 family of proteins, which includes both pro-apoptotic proteins like BAX and anti-apoptotic proteins such as BCl-2 [80]. Chronic stress-induced oxidative stress causes an imbalance between pro-apoptotic and anti-apoptotic proteins [81]. This results in the release of cytochrome c from the mitochondria into the cytoplasm [82]. Cytochrome c interacts with apoptotic protease activating factor 1 (Apaf-1) to form an apoptosome, which in turn activates caspase-9, and activated caspase-9 subsequently cleaves and activates caspase-3, leading to the dismantling of cellular structures and cell death [83]. The resulting damage to neurons due to chronic stress-induced apoptosis contributes to behavioral disorders such as anxiety [37]. Neuronal loss and dysfunction in cerebral tissue involved in emotional regulation and stress response pathways lead to alterations in behavior and mood [60]. Anxiety-related behaviors might manifest as increased vigilance, avoidance behaviors, and exaggerated responses to stressors [49]. Overall, chronic stress-induced hormonal changes lead to oxidative stress and mitochondrial dysfunction in brain tissue and trigger neuronal apoptosis and behavioral dysfunction with the systemic activation of apoptotic pathways. *S. deltoides* contains many bioactive compounds that exert protective effects against CUMS-induced apoptosis in neuronal cells (Fig. 8). In particular, the bioactive compounds in EESD play important roles in mitigating apoptosis with their antioxidant and anti-inflammatory properties [15]. Chlorogenic acid attenuates ROS generation and bolsters mitochondrial function, thereby inhibiting apoptotic pathways [84]. Rutin, on the other hand, modulates apoptotic signaling by restoring the balance between BCl-2 and BAX proteins and suppressing caspase-3 activation [85]. These compounds act complexly to preserve neuronal cell viability and prevent apoptotic cell death in response to CUMS. Furthermore, *S. deltoides* extract regulates the expression of apoptosis-related proteins to suppress CUMS-induced neuronal cell damage (Fig. 2). The extract inhibits apoptotic signaling pathways by upregulating BCl-2 expression and downregulating BAX and caspase-3 activation (Fig. 8). Thus, the various bioactive materials in EESD help alleviate neuronal cell damage caused by CUMS and promote cell survival. These bioactive compounds maintain cell survival by suppressing mitochondrial damage and intracellular ROS production, and regulate neurotransmitters such as serotonin, ultimately suppressing neuronal apoptosis and cell death.

## Conclusion

In conclusion, EESD showed a neuronal protective effect against CUMS-induced inflammatory response and hormonal imbalance. EESD with plentiful phenolic compounds protected hippocampal cells against corticosterone-induced neuronal cytotoxicity. EESD suppressed CUMS-induced anhedonia in behavior tests. EESD also ameliorated abnormal mitochondrial function related to neuronal energy metabolism. EESD regulated hypothalamic monoamine hormones by regulating cerebral stress-related indicators such as GR, CRF, ACTH, CYP11B1, and BDNF. Moreover, EESD regulated cerebral inflammatory and apoptotic protein expression levels. Ultimately, *S. deltoides* might be used as a natural plant substance to regulate CUMS-induced depression-like behaviors with the regulation of inflammatory and apoptotic signals and hormonal imbalances. In addition, it suggests that EESD may have potential therapeutic effects not only on depression but also on other diseases associated with inflammation and imbalance of the serotonin system. Therefore, it may be potentially used as a material that can affect many diseases, including depression. However, this study was primarily focused on male C57BL/6 mice to evaluate the neuroprotective effects of *S. deltoides* extract against CUMS-induced depressive-like behavior. The absence of female mice in the study is a limitation, as sex differences could influence the observed outcomes [86]. Additionally, while the study identified several bioactive compounds in EESD, it is necessary to evaluate the specific contribution of these individual compounds to the various effects. Moreover, as whole brain tissues were analyzed without regional separation, further studies should investigate specific brain regions, such as the hippocampus, amygdala, and frontal cortex, to better understand region-specific molecular changes. Future studies should aim to include female subjects, investigate additional stress-related behaviors, and further explore the role of each identified compound.

## Figures and Tables

**Table 1:** Retention time, MRM transitions and collision energy of the analytes.

Analytes	Retention time (min)	Precursor ion (*m/z*)	Product ion^1)^ (*m/z*)	Collision energy (eV)
Corticosterone	4.07	347.30	121.00	20
Norepinephrine	0.74	170.01	152.00	10
5-hydroxytryptamine (5-HT; Serotonin)	4.04	177.01	115.00	40
5-hydroxyindoleacetic acid (5-HIAA)	3.06	192.00	146.20	10
Melatonin	4.42	233.28	121.10	20

^1)^Ions are presented at *m/z* [M-H]^−^.

**Table 2:** Antibody Information used in western blot analysis.

Antibody	Catalog	Dilution used	Manufacture
β-actin	sc-69879	1:2000	Santa Cruz Biotechnology, Inc. (USA)
GR	sc-12763	1:1000	Santa Cruz Biotechnology, Inc.
CRF	sc-293187	1:1000	Santa Cruz Biotechnology, Inc.
ACTH	sc-57018	1:1000	Santa Cruz Biotechnology, Inc.
CYP11B1	sc-374096	1:1000	Santa Cruz Biotechnology, Inc.
TLR4	sc-52962	1:1000	Santa Cruz Biotechnology, Inc.
MyD88	sc-74532	1:1000	Santa Cruz Biotechnology, Inc.
p-JNK	sc-6254	1:1000	Santa Cruz Biotechnology, Inc.
p-IκB-α	sc-5404	1:1000	Santa Cruz Biotechnology, Inc.
p-NF-κB	3033S	1:1000	Cell Signaling Tech (USA)
TNF-α	sc-33639	1:1000	Santa Cruz Biotechnology, Inc.
IL-1β	sc-515598	1:500	Santa Cruz Biotechnology, Inc.
BCl-2	sc-509	1:1000	Santa Cruz Biotechnology, Inc.
Bax	sc-7480	1:2000	Santa Cruz Biotechnology, Inc.
Caspase-3	CSB-PA05689A0Rb	1:1000	Cusabio (Wuhan, China)

**Table 3:** Identification of major compounds of ethanolic extract of *Synurus deltoides*.

No.	Retention time (min)	Parents ion (*m/z*)	MS^E^ ion (*m/z*)^1)^	Compound
1	0.65	191	127, 109, 93, 85	Quinic acid
2	1.93	353	191	Chlorogenic acid
3	2.88	609	301, 300	Rutin
4	3.18	515	353, 191	1,3-Dicaffeoylquinic acid
5	3.28	615	153, 353, 191	Dicaffeoylsuccinoylquinic acid isomer

^1)^Ions are presented at *m/z* [M-H]^−^.
